# Improving explanation of motor disability with diffusion-based graph metrics at onset of the first demyelinating event

**DOI:** 10.1177/13524585241247785

**Published:** 2024-05-15

**Authors:** Michael A Foster, Ferran Prados, Sara Collorone, Baris Kanber, Niamh Cawley, Indran Davagnanam, Marios C Yiannakas, Lola Ogunbowale, Ailbhe Burke, Frederik Barkhof, Claudia AM Gandini Wheeler-Kingshott, Olga Ciccarelli, Wallace Brownlee, Ahmed T Toosy

**Affiliations:** Queen Square MS Centre, Department of Neuroinflammation, UCL Queen Square Institute of Neurology, Faculty of Brain Sciences, University College London, London, UK; Queen Square MS Centre, Department of Neuroinflammation, UCL Queen Square Institute of Neurology, Faculty of Brain Sciences, University College London, London, UK; Centre for Medical Imaging Computing, Department of Medical Physics and Biomedical Engineering, Faculty of Engineering Science, University College London, London, UK; Universitat Oberta de Catalunya, Barcelona, Spain; Queen Square MS Centre, Department of Neuroinflammation, UCL Queen Square Institute of Neurology, Faculty of Brain Sciences, University College London, London, UK; Queen Square MS Centre, Department of Neuroinflammation, UCL Queen Square Institute of Neurology, Faculty of Brain Sciences, University College London, London, UK; Centre for Medical Imaging Computing, Department of Medical Physics and Biomedical Engineering, Faculty of Engineering Science, University College London, London, UK; Queen Square MS Centre, Department of Neuroinflammation, UCL Queen Square Institute of Neurology, Faculty of Brain Sciences, University College London, London, UK; Department of Brain Repair & Rehabilitation, UCL Queen Square Institute of Neurology, Faculty of Brain Sciences, University College London, London, UK; Queen Square MS Centre, Department of Neuroinflammation, UCL Queen Square Institute of Neurology, Faculty of Brain Sciences, University College London, London, UK; Strabismus and Neuro-Ophthalmology Service, Moorfields Eye Hospital NHS Foundation Trust, London, UK; Strabismus and Neuro-Ophthalmology Service, Moorfields Eye Hospital NHS Foundation Trust, London, UK; Queen Square MS Centre, Department of Neuroinflammation, UCL Queen Square Institute of Neurology, Faculty of Brain Sciences, University College London, London, UK; Centre for Medical Imaging Computing, Department of Medical Physics and Biomedical Engineering, Faculty of Engineering Science, University College London, London, UK; Department of Radiology and Nuclear Medicine, Amsterdam UMC, Vrije Universiteit Amsterdam, Amsterdam, The Netherlands; NIHR University College London Hospitals Biomedical Research Centre, London, UK; Queen Square MS Centre, Department of Neuroinflammation, UCL Queen Square Institute of Neurology, Faculty of Brain Sciences, University College London, London, UK; Department of Brain and Behavioural Sciences, University of Pavia, Pavia, Italy; Queen Square MS Centre, Department of Neuroinflammation, UCL Queen Square Institute of Neurology, Faculty of Brain Sciences, University College London, London, UK; NIHR University College London Hospitals Biomedical Research Centre, London, UK; Queen Square MS Centre, Department of Neuroinflammation, UCL Queen Square Institute of Neurology, Faculty of Brain Sciences, University College London, London, UK; NIHR University College London Hospitals Biomedical Research Centre, London, UK; Queen Square MS Centre, Department of Neuroinflammation, UCL Queen Square Institute of Neurology, Faculty of Brain Sciences, University College London, London, UK

**Keywords:** Multiple sclerosis, MRI, diffusion MRI, clinically isolated CNS demyelinating syndrome, connectome

## Abstract

**Background:**

Conventional magnetic resonance imaging (MRI) does not account for all disability in multiple sclerosis.

**Objective:**

The objective was to assess the ability of graph metrics from diffusion-based structural connectomes to explain motor function beyond conventional MRI in early demyelinating clinically isolated syndrome (CIS).

**Methods:**

A total of 73 people with CIS underwent conventional MRI, diffusion-weighted imaging and clinical assessment within 3 months from onset. A total of 28 healthy controls underwent MRI. Structural connectomes were produced. Differences between patients and controls were explored; clinical associations were assessed in patients. Linear regression models were compared to establish relevance of graph metrics over conventional MRI.

**Results:**

Local efficiency (*p* = 0.045), clustering (*p* = 0.034) and transitivity (*p* = 0.036) were reduced in patients. Higher assortativity was associated with higher Expanded Disability Status Scale (EDSS) (β = 74.9, *p* = 0.026) scores. Faster timed 25-foot walk (T25FW) was associated with higher assortativity (β = 5.39, *p* = 0.026), local efficiency (β = 27.1, *p* = 0.041) and clustering (β = 36.1, *p* = 0.032) and lower small-worldness (β = −3.27, *p* = 0.015). Adding graph metrics to conventional MRI improved EDSS (*p* = 0.045, Δ*R*^2^ = 4) and T25FW (*p* < 0.001, Δ*R*^2^ = 13.6) prediction.

**Conclusion:**

Graph metrics are relevant early in demyelination. They show differences between patients and controls and have relationships with clinical outcomes. Segregation (local efficiency, clustering, transitivity) was particularly relevant. Combining graph metrics with conventional MRI better explained disability.

## Introduction

Diffusion-weighted MRI can be used to generate whole-brain tractography and a connectome. Its topology is described using graph theory and graph metrics, which can explain disability in multiple sclerosis (MS).^1,2^ Integration describes how networks pass information between distributed regions; segregation (or modularity) describes network organisation into smaller interconnected regions.^3^ Complex networks balance integration and segregation, giving small-worldness.^4^ Centrality assesses how well given regions (or nodes) are connected with others,^3^ and resilience is measured by how many heavily connected nodes connect to other heavily connected nodes.^5^

Long-range connections are more affected than short-range connectivity in MS. One study in early MS noted increased modularity and decreased long-range connectivity.^6^ Another identified lower global efficiency in long-range connections in established MS.^7^

Disrupted connectivity affects clinical outcomes. Damage to long-range connections is more linked to impaired cognition than short-range connections.^7^ Similarly, impaired connectivity in hippocampal networks correlates with cognitive deficits in MS.^8^ Furthermore, addition of graph metrics explains variance in disability in established MS beyond conventional MRI.^9^

The utility of adding graph metrics to conventional MRI in clinically isolated syndrome (CIS) has not been reported. Some groups looked at connectivity in isolation. One identified increased modularity and clustering in relapsing-remitting MS versus CIS and higher global and local efficiency in CIS against MS.^10^ In addition, lower node strength is reported in CIS against controls, with greater modularity associated with worse cognition.^11^ This study therefore assesses if graph analysis adds value to conventional MRI.

## Methods

### Participants

The study recruited people with symptoms suggestive of a first episode of demyelination within 3 months of presentation, identified at the National Hospital for Neurology & Neurosurgery and Moorfields Eye Hospital, both in London, UK. Age- and sex-matched healthy control (HC) participants were also recruited. Participants needed to be 18–65 years old, able to give written informed consent in English and able to have an MRI. Participants with clinically isolated syndrome (pwCIS) had CIS without prior conversion to MS, and no other condition that might affect the brain; HCs had to have no known neurological disease. The local research ethics committee approved the study protocol (13/LO/1762; 13/0231-CIS2013); all participants gave written informed consent.

### Clinical assessment

pwCIS were assessed using the Expanded Disability Status Scale (EDSS),^12^ timed 25-foot walk (T25FW), 9-hole peg test (9HPT) and the Paced Auditory Serial Addition Test (PASAT); *z*-scores were calculated for the T25FW, 9HPT and PASAT.^13^ The 2017 revisions of the McDonald criteria^14^ were used to identify MS diagnosis.

### MRI acquisition

MRI scans were acquired on a 3-Tesla Philips Achieva TX (Philips, Best, the Netherlands), upgraded during the study to a 3T Philips Ingenia CX scanner. Isotropic three-dimensional (3D) T1-weighted, fluid-attenuated inversion recovery (FLAIR), proton-density (PD) and T2-weighted scans were acquired. Multishell diffusion-weighted imaging (DWI) was acquired with 53 diffusion directions on Achieva (8 *b* = 0 s/mm^2^ images, 8 *b* = 300 s/mm^2^, 15 *b* = 711 s/mm^2^, 30 *b* = 2000 s/mm^2^) and 75 directions on Ingenia (7 *b* = 0 s/mm^2^, 20 *b* = 1000 s/mm^2^, 20 *b* = 2000 s/mm^2^, 35 *b* = 2800 s/mm^2^).

To reduce misleading differences in tractography due to movement,^15^ heads were immobilised with foam wedges and images acquired along the anterior commissure to posterior commissure line. Images were re-acquired if visual inspection demonstrated significant movement artefact.

### Lesion and tissue segmentation

Lesion masks were generated on PD images. Hyperintense lesions were outlined with the semi-automated edge-finding tool from JIM v6.0 (Xinapse Systems, West Bergholt, UK) by two experienced raters (M.A.F., S.C.). Lesions less than 2 mm in any direction were excluded, as were those in areas typical for enlarged perivascular spaces. T2-weighted and FLAIR images were references.

PD/T2- and 3D T1-weighted images were rigidly registered, and lesion masks resampled in 3D T1 space. T1-weighted images were filled using a non-local patch-match lesion-filling algorithm.^16^ These were parcellated and segmented into white matter, cortical grey matter and deep grey matter using Geodesic Information Flows^17^ following the Desikan–Killiany–Tourville parcellation protocol into 120 distinct regions.^18^ Brain tissue volumes and fractions were obtained from segmented images. Lesion proportion was calculated as a fraction of lesion volume over total intracranial volume.

### Connectome generation

DWI denoising was performed with MP-PCA;^19^ FSL 6.0 (FMRIB, Oxford, UK)^20^ ‘topup’^21^ was used for echo-planar imaging distortion correction; ‘eddy’^22^ corrected for eddy current-induced distortions and subject motion. Images were visually inspected for Gibbs ringing artefacts; affected participants were excluded. 3D T1-weighted images were co-registered to an averaged *b* = 0 s/mm^2^ image using the NiftyReg software package.^23^

Connectome generation was carried out in MRtrix3,^24^ unless stated otherwise. Five-tissue-type files were generated with FSL;^20^ brain lesions were incorporated in the white-matter mask. Nodes on parcellated images were labelled and response function calculated on corrected DWI using the ‘dhollander’ algorithm.^25^ DWI and response function files were used to extract white-matter fibre orientation distribution functions with constrained spherical deconvolution.^26^

Tractograms were formed with anatomically constrained tractography and the IFOD2 algorithm.^27,28^ A total of 30 million streamlines were generated (to facilitate production of future subnetwork connectomes) and re-weighted with SIFT2, followed by connectome generation.^29^ Connectomes were not normalised for region-of-interest volume, as the effect is ambiguous and can cause bias.^30,31^ ComBat^32^ was used to harmonise connectomes from different scanner versions; disease status (CIS or HC), age and sex were used as categorical variables to preserve biological differences. No additional inter-subject edge-weight normalisation was performed beyond SIFT2 and ComBat.^9,33–35^

### Network metrics

Graph metrics were calculated using the Brain Connectivity Toolbox^3^ for MATLAB (The MathWorks, Inc., Natick, MA, USA). Metrics were selected for different aspects of brain connectivity: integration (global efficiency), segregation (mean clustering coefficient, transitivity and mean local efficiency), centrality (mean node strength, mean betweenness centrality), resilience (assortativity coefficient) and small-worldness. Following analysis of its impact on graph metrics,^36^ thresholding was not applied to generated connectomes.

### Statistical analysis

Statistical analysis was performed with R 4.2.3.^37^ Linear regression models compared graph metrics between pwCIS and HC and associations with clinical outcomes, correcting for age and sex throughout. T25FW, 9HPT and PASAT *z*-scores were used; higher *z*-scores indicated better performance. The effect of CIS type (optic neuritis against all others) on outcomes was assessed, as were interactions of CIS type with graph metrics.

The ‘glmulti’ package^38^ was used to identify linear regression models comprising conventional MRI metrics (brain parenchymal fraction, white-matter fraction, grey-matter fraction and lesion proportion) that had associations with each measure of disability. Models were ranked by corrected Akaike Information Criterion (AICC); the lowest AICC model was selected as the ‘best’ conventional MRI model. Graph metric models were similarly determined. AICC was used for model selection to avoid over-fitting from R^2^ in isolation.^39^

Graph metrics in the best graph model were iteratively added to the best conventional MRI model to create ‘combined’ models, corrected for age, sex and CIS type. The combined model with the lowest AICC was selected as best. Model coefficients for the best conventional MRI model were compared with those for the best combined model for each measure of disability. ANOVA testing was used to calculate likelihood ratios of compared models. Significance was set at *p* < 0.05; no correction for multiple comparisons was performed.

## Results

### Descriptive statistics

**Figure 1.**
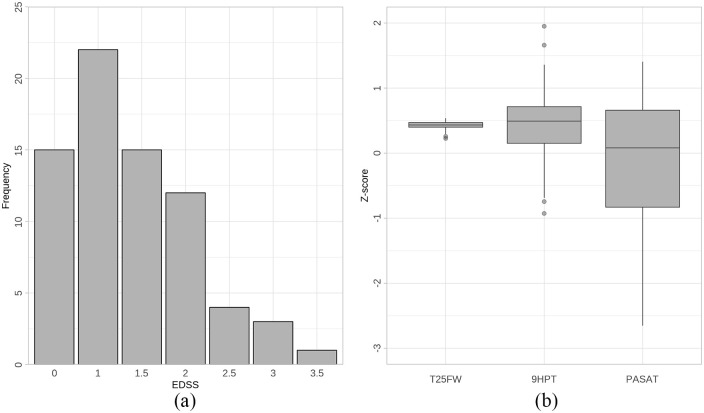
(a) Extended Disability Status Scale (EDSS) score distribution; (b) *z*-score distribution of timed 25-foot walk (T25FW), nine-hole peg test (9HPT) and Paced Auditory Serial Addition Test (PASAT).

Seventy-three people with CIS and 28 HCs were included. A total of 57 pwCIS had optic neuritis, 7 had a brainstem/cerebellum presentation, 5 presented with spinal cord symptoms, 3 had a hemispheric lesion and 1 had multifocal CIS. Mean time between symptom onset and first assessment was 59 days (standard deviation 31 days). A total of 30 pwCIS met criteria for relapsing-remitting MS^14^ at the time of their first symptoms. Further descriptives are in Table 1. Distributions of EDSS scores and T25FW, 9HPT and PASAT *z*-scores are in Figure 1.

**Table 1.** Clinical and radiological characteristics of patients and healthy controls.

	pwCIS (*n* = 73)	HCs (*n* = 28)	*p*
Age, years (SD)	31.7 (6.8)	36.3 (9.1)	0.021
Sex, number of females	44	15	0.552
Achieva/ingenia	48/25	16/12	0.440
Relapsing-remitting multiple sclerosis (%)	30 (41%)	–	–
EDSS, median (range)	1 (0-3.5)	–	–
T25FW in seconds, mean (SD)	4.68 (0.70)	–	–
T25FW *z*-score, mean (SD)	0.43 (0.06)	–	–
9HPT in seconds, mean (SD)	21.10 (2.58)	–	–
9HPT *z*-score, mean (SD)	0.42 (0.53)	–	–
PASAT score, mean (SD)	43.82 (13.11)	–	–
PASAT *z*-score, mean (SD)	-0.16 (1.13)	–	–
Number of patients whose MRI showed white-matter lesions	65	–	–
Lesion number, mean (range)	26.11 (0-121)	–	–
Lesion load, fraction of total intracranial volume (range)	0.0036 (0-0.0307)	–	–
Brain parenchymal fraction (SD)	0.763 (0.008)	0.763 (0.010)	0.9
White-matter fraction (SD)	0.308 (0.009)	0.313 (0.010)	0.033
Grey-matter fraction (SD)	0.454 (0.009)	0.450 (0.006)	0.009

pwCIS: people with CIS; HC: healthy control; SD: standard deviation; EDSS: Expanded Disability Status Scale; T25FW: timed 25-foot walk; 9HPT: 9-hole peg test; PASAT: Paced Auditory Serial Addition Test. Significant between-group differences are marked in bold.

### Differences between CIS and HC

**Figure 2.**
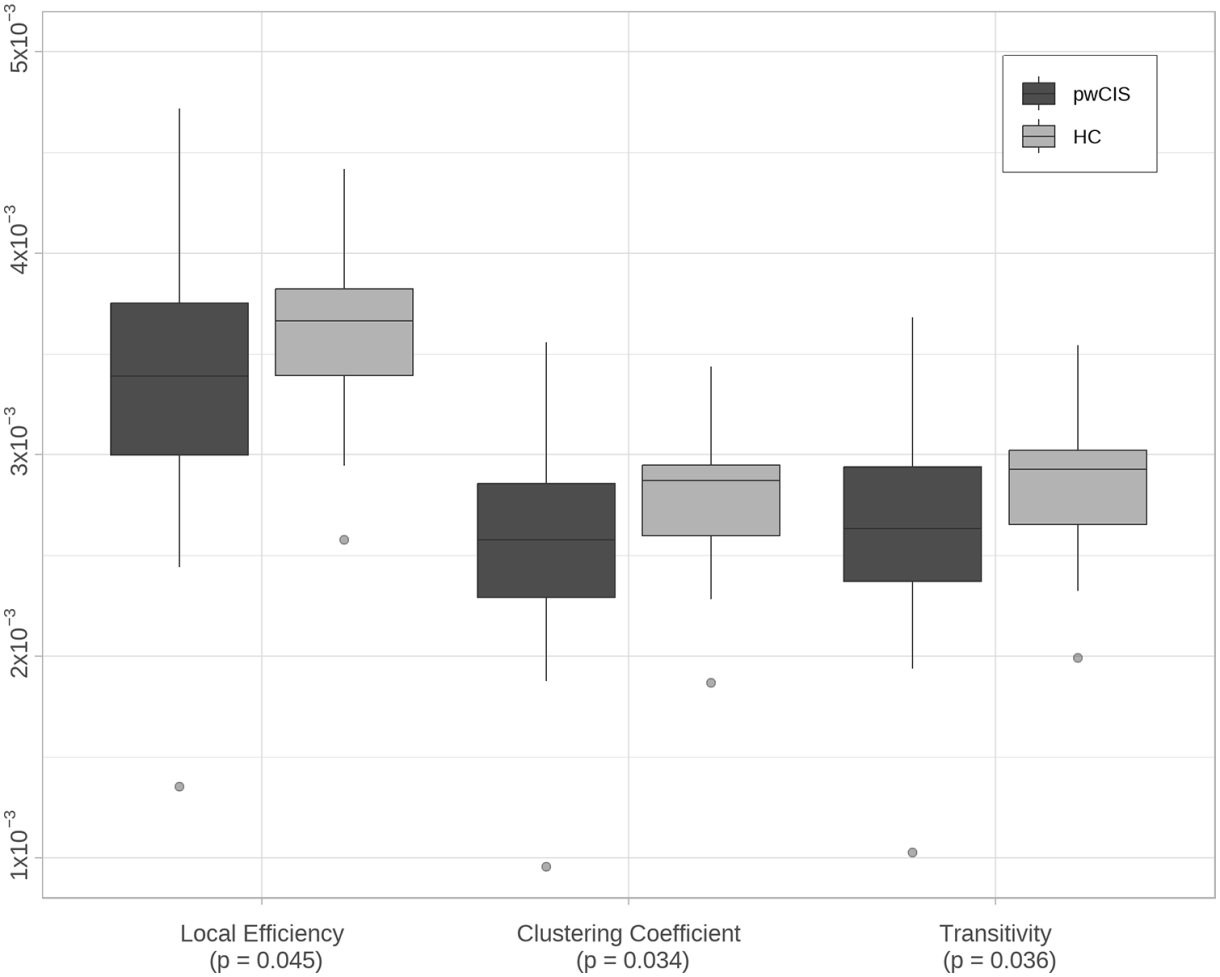
Local efficiency, clustering coefficient and transitivity in participants with CIS (pwCIS) and healthy controls (HCs).

Significant reductions in local efficiency (β = 0.25 × 10^−3^ (95% confidence interval (CI) = 0.004 × 10^−3^, 0.49 × 10^−3^), *p* = 0.045), clustering coefficient (β = 0.21 × 10^−3^ (95% CI = 0.01 × 10^−3^, 0.4 × 10^−3^), *p* = 0.034) and transitivity (β = 0.21 × 10^−3^ (95% CI = 0.01 × 10^−3^, 0.41 × 10^−3^), *p* = 0.036) were seen in pwCIS compared with HCs (Table 2 and Figure 2). No differences were noted in the other graph metrics.

**Table 2.** Graph metrics in pwCIS and HCs.

	pwCIS, mean (SD)	HCs, mean (SD)	β regression coefficient (95% CI), *p*
Global efficiency	4.05 × 10^-2^ (0.73 × 10^-2^)	4.19 × 10^-2^ (0.44 × 10^-2^)	0.16 × 10^-2^ (-0.14 × 10^-2^, 0.47 × 10^-2^), *p* = 0.291
Mean local efficiency	3.40 × 10^-3^ (0.55 × 10^-3^)	3.63 × 10^-3^ (0.43 × 10^-3^)	0.25 × 10^-3^ (0.004 × 10^-3^, 0.49 × 10^-3^) *p* **=** **0.045**
Mean clustering coefficient	2.60 × 10^-3^ (0.44 × 10^-3^)	2.79 × 10^-3^ (0.35 × 10^-3^)	0.21 × 10^-3^ (0.01 × 10^-3^, 0.4 × 10^-3^), *p* **=** **0.034**
Transitivity	2.68 × 10^-3^ (0.46 × 10^-3^)	2.87 × 10^-3^ (0.36 × 10^-3^)	0.21 × 10^-3^ (0.01 × 10^-3^, 0.41 × 10^-3^), *p* **=** **0.036**
Assortativity coefficient	-1.58 × 10^-2^ (0.30 × 10^-2^)	-1.50 × 10^-2^ (0.28 × 10^-2^)	0.007 × 10^-2^ (-0.007 × 10^-2^, 0.021 × 10^-2^), *p* = 0.300
Mean node strength	1.17 (0.19)	1.23 (0.12)	0.063 (-0.02, 0.14), *p* = 0.128
Mean betweenness centrality	462.18 (38.44)	457.86 (36.79)	-4.44 (-22.17, 13.28), *p* = 0.617
Small-worldness	1.0046 (5.27 × 10^-3^)	1.0039 (4.24 × 10^-3^)	-0.87 × 10^-3^ (-3.21 × 10^-3^, 1.47 × 10^-3^), *p* = 0.458

pwCIS: people with CIS; SD: standard deviation; HC: healthy control; CI: confidence interval.

Significant results are marked in bold.

No significant differences in graph metrics were identified between pwCIS with a confirmed diagnosis of MS and those remaining as CIS.

### Relationship of graph metrics to disability

CIS type had a weakly significant effect on EDSS (β = 0.45 (95% CI = −0.03, 0.94), *p* = 0.064) and T25FW performance (β = −0.033 (95% CI = −0.068, 0.001), *p* = 0.058); participants with optic neuritis CIS had lower EDSS scores and better T25FW times compared with non-optic neuritis CIS. It had no significant effect on 9HPT (*β* = −0.013 (95% CI = −0.318, 0.291), *p* = 0.931) or PASAT (*β* = −0.069 (95% CI = −0.739, 0.602), *p* = 0.839) outcomes.

The assortativity coefficient had a significant relationship with EDSS (β = 74.9 (95% CI = 9.1, 141), *p* = 0.026), with higher assortativity associated with increased EDSS.

For the T25FW, multiple measures of segregation had significant relationships: local efficiency (*β* = 27.1 (95% CI = 1.1, 53.1), *p* = 0.041) and clustering (β = 36.1 (95% CI = 3.1, 69.1), *p* = 0.032). Transitivity, another measure of segregation, had a weakly significant relationship (β = 32.1 (95% CI = −0.5, 64.7), *p* = 0.053). The assortativity coefficient (β = 5.39 (95% CI = 0.67, 10.11), *p* = 0.026) and small-worldness (β = −3.27 (95% CI = −5.89, −0.65), *p* = 0.015) also had significant relationships. These were all positive correlations, where increased metric value was associated with better T25FW performance, save small-worldness, which had an inverse relationship. Node strength also had a borderline significant positive relationship with the T25FW (β = 0.066 (95% CI = −0.008, 0.14), *p* = 0.085).

All measures of segregation showed trends towards positive relationships with the 9HPT: local efficiency (β = 207 (95% CI = −21, 435), *p* = 0.074), clustering (β = 267 (95% CI = −23, 557), *p* = 0.071) and transitivity (β = 264 (95% CI = −22, 550), *p* = 0.069).

No graph metrics had an association with the PASAT. There was no interaction between CIS type and graph metrics in predicting any of the disability outcomes. Results of all models tested are in Table 3.

**Table 3.** Association between graph metrics and measures of disability.

	EDSS	T25FW	9HPT	PASAT
Global efficiency	-17 (-45, 11), *p* = 0.229	1.49 (-0.51, 3.49), *p* = 0.140	11.2 (-6.4, 28.8), *p* = 0.206	0.61 (-37.6, 38.8), *p* = 0.975
Mean local efficiency	-271 (-637, 95), *p* = 0.144	27.1 (1.1, 53.1), *p* **=** **0.041**	207 (-21, 435), *p* = 0.074	49.2 (-454.4, 552.8), *p* = 0.846
Mean clustering coefficient	-326 (-794, 142), *p* = 0.167	36.1 (3.1, 69.1), *p* **=** **0.032**	267 (-23, 557), *p* = 0.071	81.6 (-559.6, 722.8), *p* = 0.8
Transitivity	-366 (-822, 90), *p* = 0.114	32.1 (-0.5, 64.7), *p* = 0.053	264 (-22, 550), *p* = 0.069	105 (-525, 735), *p* = 0.738
Assortativity coefficient	74.9 (9.1, 141), *p* **=** **0.026**	5.39 (0.67, 10.11), *p* **=** **0.026**	-9.51 (-52.41, 33.39), *p* = 0.659	-53.9 (-146.1, 38.3), *p* = 0.246
Mean node strength	-0.72 (-1.78, 0.34), *p* = 0.181	0.066 (-0.008, 0.14), *p* = 0.085	0.486 (-0.18, 1.152), *p* = 0.149	0.23 (-1.22, 1.68), *p* = 0.753
Mean betweenness centrality	0.004 (-0.001, 0.007), *p* = 0.172	-5.3 × 10^-5^ (-4.33 × 10^-4^, 3.27 × 10^-4^), *p* = 0.781	-1.42 × 10^-3^ (-4.76 × 10^-3^, 1.92 × 10^-3^), *p* = 0.397	2.19 × 10^-3^ (-5.11 × 10^-3^, 9.49 × 10^-3^), *p* = 0.550
Small-worldness	-15.6 (-54.2, 23.0), *p* = 0.421	-3.27 (-5.89, -0.65), *p* **=** **0.015**	-13 (-37.2, 11.2), *p* = 0.285	13.2 (-39.2, 65.6), *p* = 0.618

EDSS: Expanded Disability Status Scale; T25FW: timed 25-foot walk; 9HPT: nine-hole peg test; PASAT: Paced Auditory Serial Addition Test.

Results for each model reported as, in order, β regression coefficient, 95% confidence interval, and *p*-value; significant results are marked in bold.

### Addition of graph metrics to conventional MRI models

The best conventional MRI model for EDSS comprised brain parenchymal fraction and lesion proportion. For T25FW, the model contained brain parenchymal fraction, white-matter fraction, grey-matter fraction and lesion proportion. No conventional MRI models demonstrated a relationship with 9HPT or PASAT.

The best graph metric model for EDSS comprised transitivity and assortativity and the model for T25FW used global efficiency, clustering and transitivity. For 9HPT, the best model contained just transitivity; however, after correcting for age and sex, the contribution of transitivity to the model was only borderline significant (*p* = 0.069). No graph metric model demonstrated a relationship with PASAT. Details of the assessed conventional MRI and graph metric models are in Table 4.

**Table 4.** Assessment of conventional MRI and graph metric models.

Outcome measure	Metrics included	AICC	Adjusted *R*^2^ (%)
Conventional MRI metrics			
EDSS	Brain parenchymal fraction + lesion proportion	179.82	11.7
	Lesion proportion	180.63	9.2
	Grey-matter fraction + lesion proportion	180.82	10.5
T25FW	Grey-matter fraction + white-matter fraction + brain parenchymal fraction + lesion proportion	-190.10	10.0
	Grey-matter fraction + white-matter fraction + brain parenchymal fraction	-188.31	5.9
	Lesion proportion	-187.98	1.9
9HPT	Null model	117.03	0
	Grey-matter fraction	117.61	0.8
	Brain parenchymal fraction	117.98	0.3
PASAT	Null model	216.09	0
	Lesion proportion	217.34	-0.1
	Brain parenchymal fraction	217.50	-0.4
Graph metrics			
EDSS	Transitivity + assortativity	182.02	9.0
	Mean clustering coefficient + assortativity	182.11	8.9
	Transitivity + assortativity + mean node strength	182.12	10.4
T25FW	Global efficiency + mean clustering coefficient + transitivity	-195.62	15.3
	Global efficiency + assortativity	-195.44	13.5
	Assortativity + mean node strength	-195.18	13.2
9HPT	Transitivity	114.38	5.2
	Mean clustering coefficient	114.52	5.0
	Mean local efficiency	114.66	4.8
PASAT	Null model	216.09	0
	Assortativity	216.71	0.8
	Mean local efficiency + transitivity	217.52	1.4

AICC: corrected Akaike Information Criterion; EDSS: Expanded Disability Status Scale; T25FW: timed 25-foot walk; 9HPT: nine-hole peg test; PASAT: Paced Auditory Serial Addition Test.

The best three models for each outcome measure have been ranked in order of increasing AICC (lower AICC indicates better model fit).

**Figure 3.**
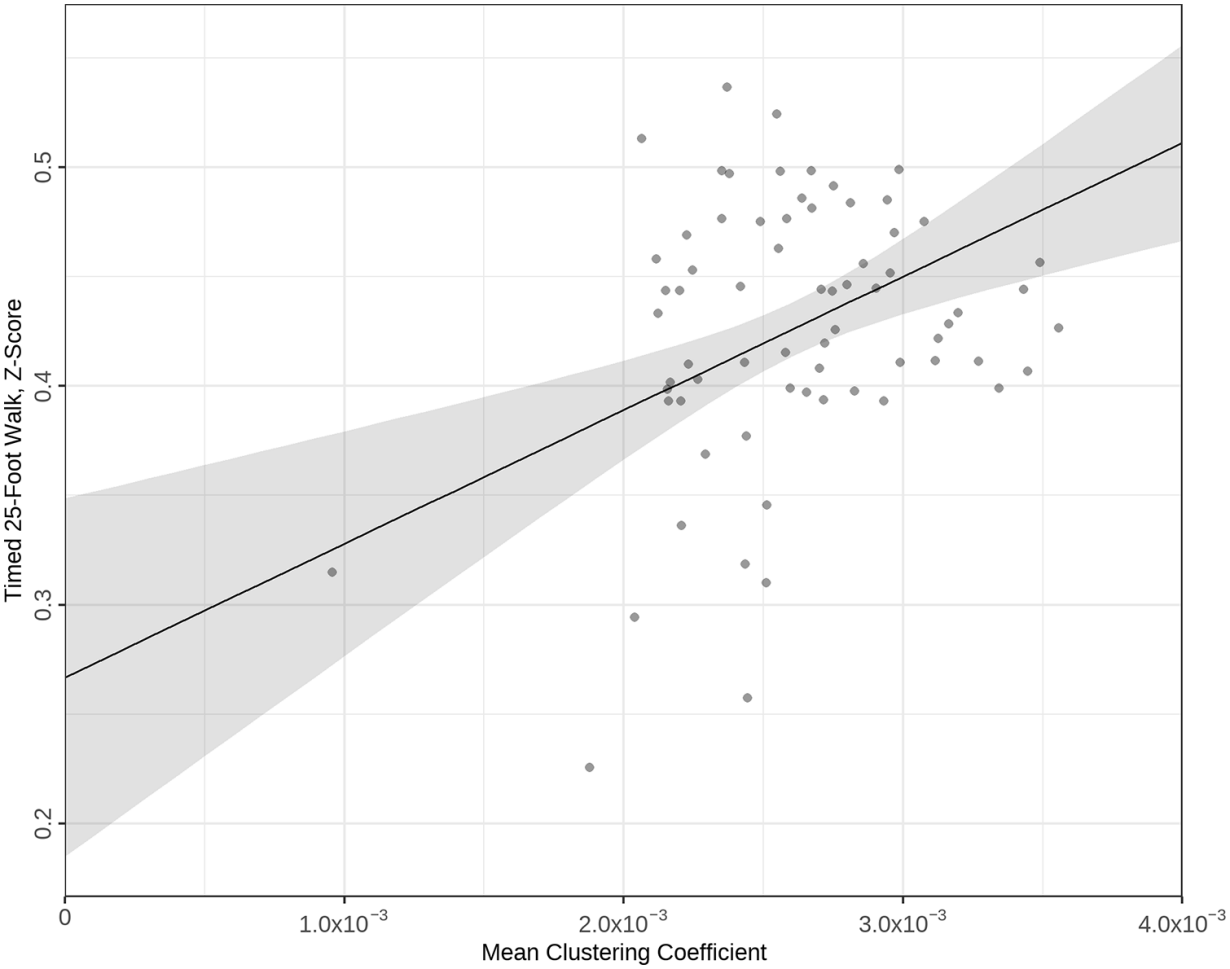
Regression plot of best timed 25-foot walk (T25FW) model comprising conventional MRI and graph metrics; T25FW *z*-scores plotted against the mean clustering coefficient.

The combined conventional and graph metric model with the best fit for EDSS added the assortativity coefficient to conventional MRI, and *R*^2^ increased from 12.6% to 16.6% (Δ = 4.0). ANOVA testing of likelihood ratios of conventional and combined EDSS models was significant at *p* = 0.045. For T25FW, the best combined model added the clustering coefficient to the conventional MRI model, increasing *R*^2^ from 11.5% to 25.1% (Δ = 13.6), *p* < 0.001. Full details of models tested are in Table 5. A regression plot for the best combined T25FW model is in Figure 3.

**Table 5.** Assessment of combined models.

Outcome measure	Best MRI model	*R*^2^ of MRI model	Added graph metrics	*p*	*R*^2^ of combined model	Δ*R*^2^
EDSS	Brain parenchymal fraction + lesion proportion	12.6%	Transitivity	0.382	12.3%	-0.3
			**Assortativity coefficient**	**0.045**	**16.6%**	**4.0**
			Transitivity + assortativity coefficient	0.085	16.5%	3.8
T25FW	Brain parenchymal fraction + white-matter fraction + grey-matter fraction + lesion proportion	11.5%	Global efficiency	0.028	17%	5.5
			**Clustering coefficient**	**<0.001**	**25.1%**	**13.6**
			Transitivity	0.001	24.1%	12.6
			Global efficiency + clustering coefficient	0.004	24%	12.5
			Global efficiency + transitivity	0.006	23%	11.5
			Clustering coefficient + transitivity	0.002	25.5%	14.0
			Global efficiency + clustering coefficient + transitivity	0.006	24.8%	13.3

EDSS: Expanded Disability Status Scale; T25FW: timed 25-foot walk.

*R*^2^ values reported are adjusted for the model and expressed as percentages. The model for each measure of disability with the lowest corrected Akaike Information Criterion value is marked in bold. *p*-values are of ANOVA comparison testing of the best conventional MRI model and best combined model.

## Discussion

One of the most notable outcomes from our analyses is the recurring role of metrics representing segregation – local efficiency, clustering coefficient and transitivity. They are all reduced in pwCIS compared with HCs. This finding is counter to those by other groups,^10,40^ where the authors identified a higher clustering coefficient in pwCIS than in HCs (other measures of segregation were not calculated). Tur et al.^11^ reported a decrease in mean node strength in pwCIS against HCs, but no differences in local efficiency or clustering coefficient.

Segregation also correlated with physical outcomes: local efficiency and clustering coefficient had a significant relationship with T25FW performance; transitivity had a borderline significant relationship. In all cases, lower graph metrics were associated with worse physical performance. Similar analyses have not previously been reported, although Tozlu et al.^41^ examined the ability of node strength to predict EDSS in an MS cohort – it was not able to discriminate between the presence or absence of significant disability. Measures of segregation also had borderline relationships with 9HPT times; that they did not reach full significance might suggest that T25FW is more sensitive to central nervous system (CNS) damage than 9HPT in early MS.

The assortativity coefficient is often described as a measure of network resilience. Brain networks, as with all biological networks, tend to be more dissortative than assortative, where high-degree nodes connect with lower-degree nodes^42^ – a characteristic also reflected by small-worldness. Assortativity increased with increasing EDSS: higher assortativity associated with higher disability. However, increased assortativity was also associated with improved T25FW, indicative of better lower-limb function: higher assortativity correlated with worse EDSS and better T25FW.

This apparent paradox could be explained by examining both the range of outcomes of the two scores and the outcome scoring tools themselves. In our cohort (Figure 1), T25FW *z*-scores all fall above 0, indicating better performance than an MS reference population. Although 15 pwCIS had an EDSS of 0 (21% of the cohort), the majority had measurable disability on EDSS. The T25FW is sensitive to variation in lower-limb function in early MS (even where performance is good), but the EDSS only measures pathological disability. The relationship of assortativity with EDSS is therefore predominantly a relationship with disability, whereas its relationship with T25FW is to values that would be considered better than a typical MS population. However, it should be noted that T25FW *z*-scores were calculated with reference to the National MS Society data set, rather than an age- and sex-matched data set; our cohort is likely younger than the comparison data set with shorter disease duration, which may explain the better-than-average T25FW performance.

Furthermore, the two scores measure disability differently – EDSS is a composite score derived from multiple systems, whereas the T25FW is a single, specific measurement of lower-limb function. Higher EDSS-measured disability (4.0 and above) is heavily influenced by ambulation, but it hardly features in lower levels (the highest EDSS in the study was 3.5).^12^ In addition, as optic neuritis is highly represented in our cohort, the main contributor to EDSS was likely visual disability. Indeed, in a post hoc analysis, the relationship was preserved after adding T25FW as a model co-variate, supporting the conclusion that EDSS in our cohort was driven by non-motor disability; similarly, there was good correlation between EDSS and LogMAR (Pearson’s coefficient 0.446, *p* < 0.001), but no correlation between EDSS and T25FW scores. This suggests that the relationship of EDSS with assortativity mostly relates to visual disability. Notably, there were no significant relationships of visual outcome scores with graph metrics. It may be that a visual injury causes a maladaptive increase in assortativity, but an actual loss of assortativity is directly connected with impaired lower-limb function.

Small-worldness also correlated with T25FW outcomes: higher small-worldness was associated with poorer lower-limb function. Since small-worldness reflects the balance of integration and segregation,^4^ this may simply be a consequence of the relationship of lower measures of segregation with poorer T25FW outcomes. Indeed, small-worldness and assortativity measure similar characteristics; a network with low assortativity will have high small-worldness. These metrics were strongly negatively correlated in our cohort (post hoc analysis: Pearson’s −0.707, *p* < 0.001).

Global efficiency, an indicator of integration, was not affected in pwCIS compared with HCs nor did it correlate with outcome measures. Other studies of structural connectivity in early CIS do not report results for global efficiency, nor the related characteristic path length.^3^ However, there are multiple reports of reductions in global efficiency in established MS when compared with HCs.^7–9,43^ Charalambous et al.^9^ also noted that reduced global efficiency was associated with higher EDSS in people with MS. This may suggest that global efficiency is preserved early in the disease course – the brain is able to adapt to low burdens of injury, but after a certain threshold loses this capacity, with resulting impact on its ability to distribute signals throughout the network. On the contrary, smaller insults may cause interruptions to localised network connections which are more difficult to overcome, causing network subunit breakdown and loss of segregation.

This work provides conclusive evidence that graph metrics improve our ability to explain disability beyond conventional MRI and does so for the first time in people at onset of their first demyelinating event. It extends previous findings^9^ that the addition of graph metrics to conventional MRI can improve the ability of linear regression models to explain variance in physical outcomes: the ability of the EDSS model to predict variance improved from 12.6% to 16.6% (a change of 4%) and the T25FW model *R*^2^ was improved by 13.6 from 11.5% to 25.1%.

The inability of any metric, conventional or graph, to associate with the PASAT is also important. The finding that conventional MRI is unable to explain PASAT outcomes confirms there is a mechanism of disability that is not structural (e.g. brain volumes or lesion proportion). While whole-brain graph metrics also lacked a relationship, analysis of cognitive subnetworks may reveal a stronger association. The findings may also suggest cognitive performance is largely preserved in early MS.

One limitation of the study is that MRI data were acquired on two different scanner versions, with different multishell DWI acquisition protocols. We have mitigated this by use of the ComBat algorithm^32^ – although originally developed for genomic work, its use in DWI is validated across multiple conditions and patient groups.^44,45^

As noted above, correction for multiple comparisons was not performed. As some of the analysis was exploratory, the conventional Bonferroni approach was too conservative and assumed that the tests were independent (which was not the case in this study).^46^ Where there is biological similarity in the relationships assessed by individual tests, a biological pattern of moderately significant results is very unlikely to occur by chance. In this context, it was important to highlight the patterns and interrogate biologically isolated individual results.

In addition, the analysis is of people in the very early stages of disease. Levels of disability are low, and the range of physical outcomes is narrow. It is possible that with greater disease duration, more differences in disability may emerge and subsequently more associations with graph metrics. Similarly, as this is a cross-sectional study, it is not yet possible to say if these associations will be sustained through the disease process. Longitudinal data are being collected, which will delineate the predictive value of graph metrics.

In conclusion, these analyses demonstrate the utility of graph metrics derived from multishell DWI-based structural connectomes in the assessment of motor function early in CIS. They show how impaired segregation may be an early indicator of poorer motor function. Furthermore, we show how the addition of graph metrics to conventional MRI can improve the performance of models of motor (particularly lower-limb) function. Future analyses will examine the contribution of graph metrics based on brain subnetworks, as well as establish if these findings are persistent longitudinally and whether graph metrics can predict long-term outcomes.
